# The Role of Quantitative Aortographic Assessment of Aortic Regurgitation by Videodensitometry in the Guidance of Transcatheter Aortic Valve Implantation

**DOI:** 10.5935/abc.20180139

**Published:** 2018-08

**Authors:** Yosuke Miyazaki, Rodrigo Modolo, Mohammad Abdelghani, Hiroki Tateishi, Rafael Cavalcante, Carlos Collet, Taku Asano, Yuki Katagiri, Erhan Tenekecioglu, Rogério Sarmento-Leite, José A. Mangione, Alexandre Abizaid, Osama I.I. Soliman, Yoshinobu Onuma, Patrick W. Serruys, Pedro A. Lemos, Fabio S. de Brito Jr.

**Affiliations:** 1 Department of Cardiology - Thoraxcenter, Erasmus Medical Center Rotterdam, Rotterdam – Netherlands; 2 Department of Cardiology - the Academic Medical Center - University of Amsterdam, Amsterdam – Netherlands; 3 Departamento de Medicina Interna - Divisão de Cardiologia - Universidade de Campinas (UNICAMP), Campinas, SP – Brazil; 4 Division of Cardiology - Department of Clinical science and Medicine - Yamaguchi University Graduate School of Medicine, Ube, Yamaguchi – Japan; 5 Instituto de Cardiologia do Rio Grande do Sul/Fundação Universitária de Cardiologia e Universidade Federal de Ciências da Saúde de Porto Alegre, Porto Alegre, RS – Brazil; 6 Hospital Beneficência Portuguesa de São Paulo, São Paulo, SP – Brazil; 7 Instituto Dante Pazzanese de Cardiologia, São Paulo, SP – Brazil; 8 Cardialysis, Rotterdam – Netherlands; 9 NHLI, Imperial College London, London – United Kingdom; 10 Hospital Israelita Albert Einstein, São Paulo, SP – Brazil; 11 Instituto do Coração (InCor), Faculdade de Medicina da Universidade de São Paulo, São Paulo, SP – Brazil

**Keywords:** Aortic Valve Insufficiency/diagnostic imaging, Angiography/evaluation, Heart Valve Prosthesis Implantation, Transcatheter Aortic Valve Replacement

## Abstract

**Background:**

Balloon post-dilatation (BPD) is often needed for optimizing transcatheter heart valve (THV) implantation, since paravalvular leak (PVL) after transcatheter aortic valve implantation is associated with poor outcome and mortality. Quantitative assessment of PVL severity before and after BPD is mandatory to properly assess PVL, thus improving implantation results and outcomes.

**Objective:**

To investigate a quantitative angiographic assessment of aortic regurgitation (AR) by videodensitometry before and after BPD.

**Methods:**

Videodensitometric-AR assessments (VD-AR) before and after BPD were analysed in 61 cases.

**Results:**

VD-AR decreased significantly from 24.0[18.0-30.5]% to 12.0[5.5-19.0]% (p < 0.001, a two-tailed p < 0.05 defined the statistical significance). The relative delta of VD-AR after BPD ranged from -100% (improvement) to +40% (deterioration) and its median value was -46.2%. The frequency of improvement, no change, and deterioration were 70% (n = 43), 25% (n = 15) and 5% (n = 3), respectively. Significant AR (VD-AR > 17%) was observed in 47 patients (77%) before and in 19 patients (31%) after BPD.

**Conclusions:**

VD-AR after THV implantation provides a quantitative assessment of post-TAVI regurgitation and can help in the decision-making process on performing BPD and in determining its efficacy.

## Introduction

Balloon post-dilatation (BPD) is often needed for optimizing transcatheter heart valve (THV) implantation, since paravalvular leak (PVL) after transcatheter aortic valve implantation (TAVI) is associated with long- term fatal prognosis.^1^^-^^5^ The incidence of moderate or severe PVL following TAVI varies from 0% to 24% and that of mild PVL from 7% to 70%.^6^ BPD is performed in 21% to 28% of cases with the first generation THVs.^7^^,^^8^ Although newer generations of THVs have been designed to reduce the PVL, BPD is still performed in up to 17% of cases receiving the new generation of THVs.^7^^,^^9^^,^^10^ Therefore, BPD remains one important technique to optimize implantation of the THV.

The Valve Academic Research Consortium-2 (VARC-2) consensus document recommends to perform quantitative and semi-quantitative hemodynamic assessments of PVL severity and other definitions for valve failure than mild PVL only.^11^ TAVI under conscious sedation is increasingly adopted in clinical practice (i.e. the minimalist approach) restricting the usage of transesophageal echocardiography (TEE) as a guidance for TAVI and increasing the role of aortography as a screening tool to determine the severity of PVL during the procedure. We have previously reported the *in vitro* and *in vivo* validation of quantitative angiographic assessment of aortic regurgitation (AR) by videodensitometry technique after implantation of THV with an excellent reproducibility and accuracy.^12^ This technique provides an accurate assessment of the severity of PVL and it has been shown that a Videodensitometric-AR (VD-AR) > 17% correlates with increased mortality and impaired reverse cardiac remodelling as determined by echocardiography after TAVI.^13^^,^^14^ This prognostic cut-off value (VD-AR > 17%) could have the potential to guide operators in deciding the need for BPD. However, the change of VD-AR from before to after BPD has not been investigated. The aim of this study is to assess a quantitative aortographic approach of PVL by videodensitometry before and after BPD.

## Methods

### Study design

**Figure 1:**
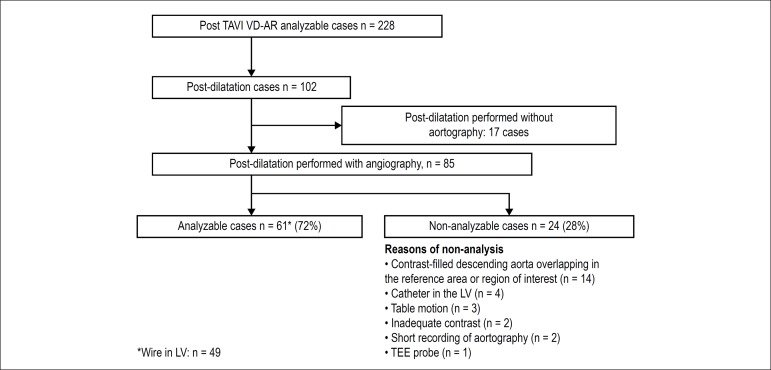
Flowchart of this study. TAVI: transcatheter aortic valve implantation; VD-RA: Videodensitometric of aortic regurgitation; TEE: transesophageal echocardiography.

This is a report on patients enrolled in the the Brazilian TAVI registry including between January 2008 and January 2013. List of participating centers, inclusion and exclusion criteria and technical description of TAVI-procedure were previously reported.^15^ The study protocol was approved by the ethics committee at each of the participating centers and all patients provided informed written consent. Three hundred ninety-nine patients were enrolled in the Brazilian TAVI registry in that period. VD-AR was performed and found to be analysable in 228 patients.^16^ In this population, 102 patients underwent BPD, and in 17 cases, no angiography was available before BPD. Out of 85 cases with available aortograms before and after BPD, VD-AR was analysable at both time points in 61 cases (Figure 1). The reasons of non-analysable are discriminated in Figure 1.

### Aortographic assessment of AR

Aortic root angiography was performed before and after BPD, using at least 20 ml of non-ionic contrast injected through a pigtail catheter positioned above the prosthetic valve stent (in case of a balloon-expandable device) or within the distal third of the prosthetic valve stent (in case of a self-expanding device). The decision on the total contrast volume and speed of injection, catheter size, and the projection were left to the discretion of the operators. Visual assessment of AR was performed by experienced observers based on Sellers’ grade.^17^ In a blinded fashion, assessment of post-BPD aortograms was performed by observers different from those who analyzed pre-BPD aortograms.

### Quantification of AR using videodensitometric technology

**Figure 2:**
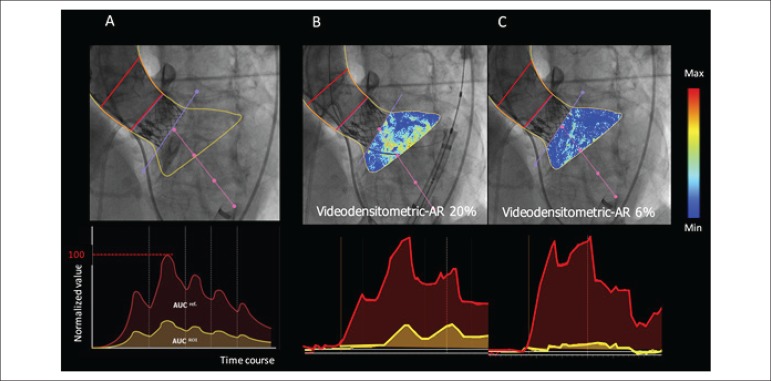
Videodensitometric assessment of aortic regurgitation. A) Delineation of the aortic root (reference region: red area in the aortography) and the subaortic one third of LV (ROI: yellow area in the aortography) are shown by the analyser. The time-density curves are provided for both ROI (yellow) and reference (red) regions, and the AUC is automatically computed by the software time-density integrals. VD-AR corresponds to the relative AUC, which is automatically calculated as the ratio of the relative AUC in the ROI (yellow) to that in the reference area (red). Theoretically, the value of VD-AR ranges from 0 to 1. B) An example of VD-AR measurement before BPD. C) An example of VD-AR measurement after BPD. Reproduce and adopted from Tateishi et al. EuroIntervention 2016^14^

VD-AR before and after BPD was analysed at an independent core laboratory (Cardialysis Clinical Trials Management and Core Laboratories, Rotterdam, the Netherlands) by experienced observers using a dedicated software (CAAS A-Valve 2.0.2; Pie Medical Imaging, Maastricht, The Netherlands). The details of this technique have been described elsewhere.^12^^-^^14^^,^^16^^,^^18^ After drawing the contours of the aortic root (*i.e*. reference region) and the subaortic one third of the left ventricle (i.e. region of interest [ROI]), the contrast time-density curves were generated for both regions over at least three cardiac cycles after contrast injection. The areas under these curves (AUC) are automatically calculated and represent the time-density integral. VD-AR is automatically calculated as the ratio of the AUC of the ROI to that of the reference region (Figure 2). Theoretically, the value of VD-AR ranges from 0.0% to 100%. The relative delta VD-AR was calculated as = (VD-AR after BPD - VD-AR before BPD)/VD-AR before BPD, where a negative value indicates an improvement of the severity of AR.

### THV and post-dilatation balloon diameters / annulus diameter ratios

Multislice computed tomography (MSCT) was performed following the local radiological protocol. Cover index was calculated as “(prosthesis nominal diameter – annulus diameter) / (prosthesis nominal diameter) × 100”. The post-dilatation balloon size / annulus diameter ratio was calculated as “(balloon nominal diameter – annulus diameter) / (balloon nominal diameter) × 100”.

### Statistics

When continuous variables were normally distributed, we summarized data as mean ± standard deviation.^19^ If they were not normally distributed, median and inter-quartile range [IQR] were used. Mann-Whitney test was used to compare continuous variables between independent samples. Wilcoxon signed ranks test was performed to compare the serial changes between before and after BPD. All analyses were performed with SPSS 23 (IBM, Armonk, NY, USA). A two-tailed p < 0.05 defined the statistical significance.

## Results

Baseline characteristics and echocardiographic data of this population (n = 61) are shown in Table 1. The mean age was 81.6 ± 7.6 years, and patients had a high Society of Thoracic Surgeons (STS)-Predicted Risk Of Mortality score, 8.8(4.6-16.3). Either CoreValve (Medtronic, Minneapolis, MN, USA) (72%) or SapienXT (Edwards Lifesciences, Irvine, CA, USA) (28%) have been implanted. In most cases, TAVI was performed with general anaesthesia (98%) and transfemoral approach (97%).

**Table 1:** Baseline and echocardiographic characteristics of the study population (n = 61)

Variables	Median (IQR)/Frequency
**Clinical characteristics**	
Age, years (median[IQR])	81.6 ± 7.6
Male gender, n (%)	37(60.7)
BMI, kg/m^2^	24.6 ± 3.9
NYHA II, n (%)	13(21.3)
NYHA III, n (%)	27(44.3)
NYHA IV, n (%)	21(34.4)
Hypertension, n (%)	47(77.0)
DM, n (%)	15(24.6)
Renal insufficiency*, n (%)	51(83.6)
CAD, n (%)	31(50.8)
PAD, n (%)	13(21.3)
COPD, n (%)	15(24.6)
PH**, n (%)	12(19.7)
Prior PCI, n (%)	15(24.6)
Prior CABG, n (%)	10(16.4)
Prior MI, n (%)	6(9.8)
Prior stroke, n (%)	6(9.8)
Prior BAV, n (%)	4(6.6)
Prior AVR, n (%)	1(1.6)
Prior PMI, n (%)	7(11.5)
Af/AFL, n (%)	9(15.0)
STS-PROM, %	8.8[4.6-16.3]
EuroSCORE, %	15.9[9.2-25.4]
**Preprocedural echocardiographic parameters**	
LVDd, mm	50.0[46.0-55.0]
LVEF,	61.0[45.0-68.0]
LVM index, %	136.9[114.2-162.9]
AVA, cm^2 ^	0.6[0.5-0.8]
Peak PG, mmHg	75.0[64.0-92.5]
Mean PG, mmHg	47.0[41.0-61.0]
MR >mild, n (%)	16(26.2)
TEE guidance, n (%)	56(91.8)
General anesthesia, n (%)	60(98.4)
Transfemoral approach, n (%)	59(96.7)
**Procedural characteristics**	
CoreValve, n (%)	44(72)
CoreValve 26mm, n (%)	9(20.5)
CoreValve 29mm, n (%)	17(38.6)
CoreValve 31mm, n (%)	18(40.9)
Sapien-XT, n (%)	17(28)
Sapien-XT 23mm, n (%)	7(41.2)
Sapien-XT 26mm, n (%)	8(47.1)
Sapien-XT 29mm, n (%)	2(11.8)
Pre-dilatation performed, n (%)	18(29.5%)

BMI: body mass index, NYHA: New York Heart Association, DM: diabetes mellitus, CAD: coronary artery disease, PAD: peripheral artery disease, COPD: chronic obstructive pulmonary disease, PH: pulmonary hypertension, PCI: percutaneous coronary intervention, CABG: Coronary artery bypass grafting, MI: myocardial infarction, BAV: balloon aortic valvuloplasty, AVR: aortic valve replacement, PMI: pacemaker implantation, AF: atrial fibrillation, AFL: atrial flutter, STS-PROM: the Society of Thoracic Surgeons - predicted risk of mortality, LVDd: left ventricular diastolic diameter, LVEF: left ventricular ejection fraction, LVM index: left ventricular mass index, AVA: aortic valve area, PG: pressure gradient, MR: mitral regurgitation, TEE: transesophageal echocardiography.

* Defined as glomerular filtration rate < 60 mL/min,

** Defined as a systolic pulmonary artery pressure ≥ 60 mm Hg at rest

### Influence of BPD on VD-AR

**Figure 3:**
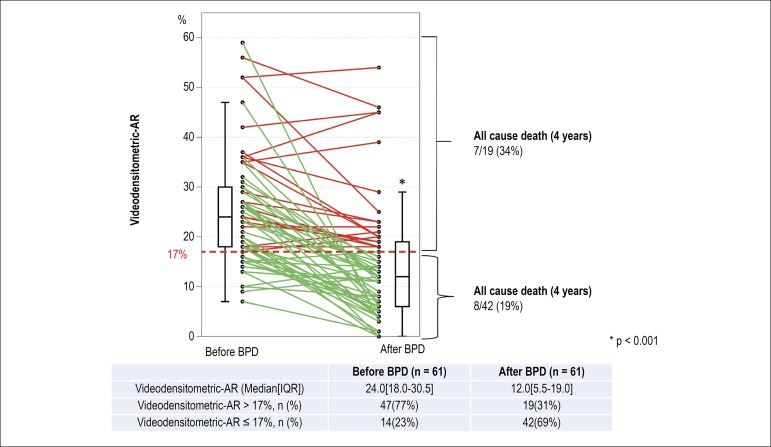
Serial changes of the Videodensitometric-AR. Individual serial changes before and after balloon post-dilatation are shown in this figure. In patients with VD‑AR > 17%, 7 deaths (34%) occurred, whereas in patients with VD-AR ≤ 17%, 8 deaths (19%) were observed.

**Figure 4:**
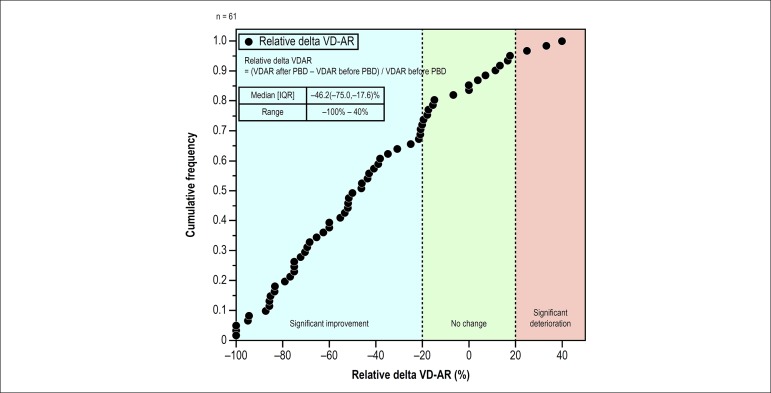
Cumulative frequency curve of the rate of improvement or deterioration of aortic regurgitation by balloon post-dilatation. The formula of the relative delta VD-AR was “(VD-AR after BPD - VD-AR before BPD)/VD-AR before BPD”. Negative values indicate improvements of AR after BPD, whereas positive values stand for deterioration of AR after BPD. Using twenty-fifth percentile of the absolute delta VD-AR, arbitrarily we defined a relative delta of less than -20% as a significant improvement (blue), from -20 to 20% as no change (green), and 20% more as a significant deterioration (orange).

The change of VD-AR from before- to after- BPD is shown in Figure 3 and a representative case is displayed in Figure 2 and Movie 1. VD-AR decreased significantly from 24.0[18.0-30.5]% (before BPD) to 12.0[5.5-19.0]% (after BPD) (p < 0.001). The median value of absolute delta VD-AR was -10.0%, corresponding to a relative delta of - 46.2% (range: -100% to +40%). The frequencies of any improvement or deterioration of AR (as defined by VD-AR) were 82% (n = 50) and 18% (n = 11), respectively (Figure 4). The 25th percentile of the relative delta VD-AR was 20%, and this cut-point was arbitrarily used to define “a significant change” as follows: a relative delta < -20% defined as “a significant improvement”, a relative delta of -20 to +20% as “no change”, and a relative delta > +20% as “a significant deterioration”. There were 43 patients (70%) with significant improvement, 15 patients (25%) with no change, and 3 patients (5%) with significant deterioration.

**Video 1:** Videodensitometric assessment of aortic regurgitation before and after balloon post-dilatation. Left panel shows VD-AR assessment before BPD (VD‑AR = 20%). Right panel shows VD-AR assessment after BPD (VD-AR = 6%).

The THV cover index and the balloon size used in post-dilatation were both available in 38 out of 61 patients – 25 (66%) among those with significantly improvement of PVL after BPD, 11 (29%) with no change in VD-AR, and 2 (5%) with deterioration of AR. THV cover index was 11.5[4.1,15.9] and ranged from 0.0% to 22.8% in patients with a significant improvement of AR, and 13.8[3.3,16.5], ranging -29.0% to 19.3% in those with no change or a significant deterioration of AR. Post-dilatation balloon size / annulus diameter ratio was 0.0[-7.9,7.6] and ranged from -25.0% to 14.3% in patients with a significant improvement of AR, and 0.0[-5.6,13.4], ranging from -33.3% to 16.4% in patients with no change or with a significant deterioration of AR.

### Serial change of AR based on Sellers’ grade

**Figure 5:**
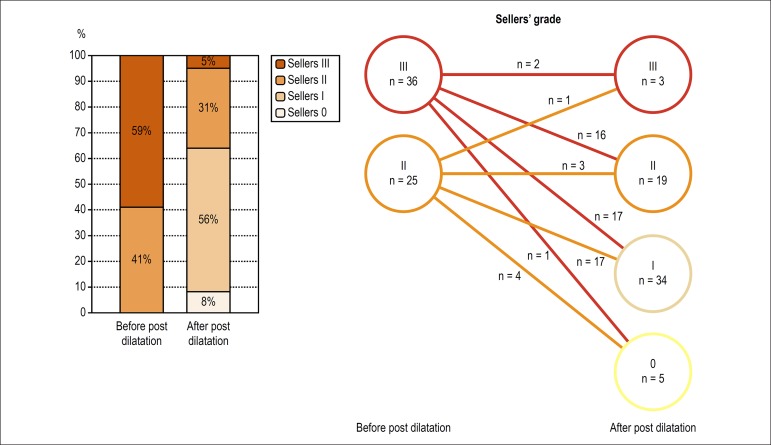
Serial changes of visual aortographic assessment.

Before BPD, AR was visually classified as Sellers’ III in 36 patients (59%), and as Sellers’ II in 25 patients (41%). After post-dilatation, there were 3 (5%) cases with Seller’ III, 19 (31%) cases with Sellers’ II, 34 (56%) cases with Sellers’ I and 5 (8%) cases with Sellers’ 0. Out of 36 patients with Sellers’ III before BPD, 34 patients had their Sellers’ grade reduced (to Sellers’ II in 16, Sellers’ I in 17, and Sellers’ 0 in one patient. Out of 25 patients with Sellers’ II before BPD, PVL improved to Sellers’ I in 17 patients and to Sellers’ 0 in 4 patients, deteriorated to Sellers’ III in one patient, and remained unchanged (Sellers’ II) in three patients (Figure 5).

### Efficacy of BPD

**Figure 6:**
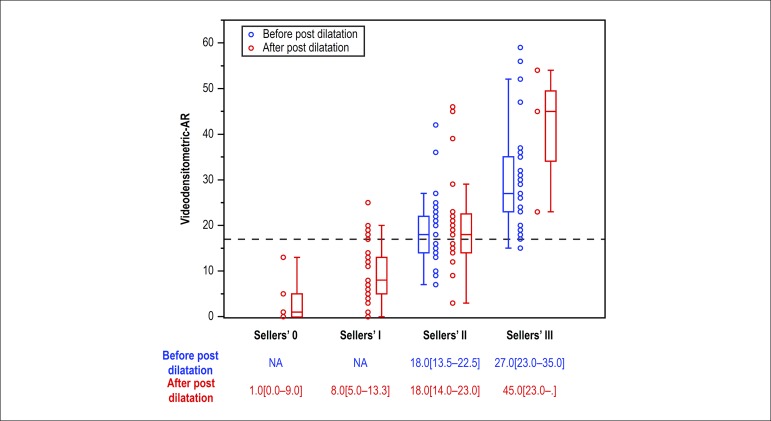
Videodensitometric- assessment of aortic regurgitation and Sellers’ grade before and after balloon post-dilatation.

Before BPD, VD-AR > 17%, a value that has a prognostic significance in long-term follow-up, was observed in 47 patients (77%). Fourteen cases (23%) had a VD-AR ≤ 17%, eleven (11/14, 79%) were evaluated as Sellers’ II before BPD and 3 (3/14, 21%) as Sellers’ III. After BPD, VD-AR > 17% was observed in 19 patients (falling from 77% to 31% of subjects) – 3 patients (16%) in Seller’s III, 10 patients (53%) in Sellers’ II, and 6 patients in Sellers’ I (32%) (Figure 6). In addition, in these patients with VD-AR > 17%, 7 deaths (34%) occurred during follow-up period, whereas among 42 patients with VD-AR ≤ 17%, 8 patients (19%) died.

Predilatation was performed in 18 patients and had no impact on the reduction of AR assessed by VD-AR. VD-AR was 25.5% (19.5%-36.0%) with predilatation and 23.0% (16.0%-29.0%) without predilatation (p = 0.159) before PBD, and 16.5% (9.5%-22.8%) with predilatation and 11.0% (5.0%-17.0%) without predilatation (p = 0.106) after PBD. Normalized delta VD-AR was -44.5 (-60.1 – -13.0) with predilatation and -50.0(-75.0 – -17.9) without predilatation (p = 0.569).

## Discussion

This is the first study to report the value of VD-AR in assessing periprocedural changes in AR. In clinical practice, echocardiogram and aortography are the standard tools to define the device success. As mentioned in the Valve Academic Research Consortium-2 (VARC-2) consensus document, quantitative and semi-quantitative hemodynamic assessment are recommended to assess AR severity by echocardiogram and moderate-to-severe AR is defined as valve failure.^11^^,^^20^

Nombela-Franco et al.^8^ reported serial changes using semi-quantitative grading based on echocardiogram and showed a reduction of at least 1 degree of AR in 71% of patients. To make a decision whether BPD is needed or not, echocardiogram is an important tool to evaluate the severity of AR. However, we must consider that with the increasing minimalist TAVI approach, the usage of TEE as a guidance of TAVI is becoming unfeasible. Moreover, low inter-observer agreement for the PVL 4-class grading (kappa 0.481) and the 7-class grading (kappa 0.517) has been reported,^21^ making a more reliable technique necessary.^18^ These facts support the value of aortography with VD assessment as the most practical and objective screening tool to determine the severity of PVL during the procedure. The technique has a median time of execution of 3 minutes.

We have previously shown that a VD-AR > 17% correlates with increased mortality and with impaired cardiac reverse remodelling as determined by echocardiography after TAVI with excellent reproducibility.^13^^,^^14^ This value (VD-AR > 17%) could be decisive in helping the operator to make a decision as whether BPD should be performed during the procedure. When BPD was performed, we showed that before BPD, 77% of patients had a VD-AR > 17%, and the other patients (VD-AR ≤ 17%) (23%) would not require BPD. This finding is important, since BPD is associated with higher rate of cerebrovascular events compared to the patients without BPD.^8^^,^^22^ Avoiding unnecessary BPD would possibly reduce the risk of cerebrovascular events as well as procedural costs. Moreover, most cases of VD-AR ≤ 17% before BPD were found in Sellers’ II, suggesting that the visual assessment of the Sellers’ classification could lead to unnecessary PBD.

After BPD, VD-AR > 17% was still seen in 31% of patients. Based on current available data, for patients with residual AR (VD-AR > 17%), additional measures should be taken. We found higher mortality in patients with VD-AR > 17% compared to patients with VD-AR ≤ 17% during the follow-up (34% vs. 19%). Although the difference in mortality was not significant (log rank p = 0.273) in this small population with BPD, a tendency for high mortality was previously reported in patients with VD-AR > 17% in a large population.^14^^,^^16^

VD-AR deteriorated numerically in 11 patients, and this deterioration was significant in 3. This comes in agreement with previous studies which also reported AR deterioration in a small proportion of patients after BPD,^8^ and could be due to prosthetic overexpansion with secondary leaflet maladaptation and transvalvular regurgitation.^23^

Serial changes of VD-AR showed predominantly improvement of AR. A reduction of the regurgitation by BPD was reported in 68%-91% in the literature.^8^^,^^24^ The mechanisms of regurgitation after implantation of THV are multifactorial as, for example, calcification of the native aortic annulus and left ventricular outflow tract (LVOT) and cover index are well known predicting factors of regurgitation after implantation of a THV.^19^^,^^26^^-^^34^

To make a decision whether BPD is needed or not and to judge its efficiency, repeated injections of large doses of contrast medium would be needed. Contrast medium volume used in this population was 150[131-209] ml/procedure. In the setting of TAVI, peri-procedural acute kidney injury (AKI) develops in 12% to 57% of cases and portends a significant increase in early and late mortality.^34^^,^^35^ The mechanisms of AKI following TAVI are multifactorial, and the role of the contrast medium volume is controversial.^36^ However, there is some evidence suggesting that a larger contrast volume is related to an increased risk of AKI after TAVI.^34^^,^^37^ Taking into account the important role of aortography in the minimalist TAVI era, repeated aortograms cannot be avoided. However, the possibility of reducing contrast medium is reported using a diastolic phase-synchronized injection of only 8 ml of contrast medium in an *in-vitro* setting.^12^ This technique could enable the reduction of the total amount of contrast medium during the procedure.

### Limitations

After implantation of the THV, the guidewire is frequently left in the left ventricle and may produce artificial transvalvular regurgitation.^38^ However, the effect of the guidewire on AR during TAVI is variable according to the weight of the wire. Most operators decide whether to perform BPD with or without a guidewire in LV by using echocardiography and aortography. Indeed, in the present study, VD-AR before BPD was analysed either with (n = 49) or without (n = 12) the guidewire being left in the left ventricle.

One limitation of our study is the absence of data on aortic regurgitation index, thus lacking the possibility of comparing this to our method. Limitations of VD-AR assessment are its feasibility. The current report is a retrospective study so that the acquisition of aortography was not dedicated for VD-AR assessment. In order to perform videodensitometric assessment appropriately, the acquisition of aortography should be done without overlapping ROI with contrast filled ascending/descending aorta. Recently, Teng et al.^39^ reported how to plan an overlap free projection for VD-AR assessment.^39^ A dedicated acquisition protocol would achieve a high feasibility of assessment. We tried to overcome this limitation by choosing the cases that did had an adequate acquisition of images, lowering our sample size. However, a prospective clinical study is needed to confirm this hypothetical assumption. So far, CAAS-A-valve software is available as an offline system. Currently, attempts are being made to allow online assessment.^40^ In the near future, online system will probably foster the VD-AR as guidance for TAVI.

In this registry, no echocardiographic parameters recorded were reported after THV deployment but before BPD. The information of calcification of the native aortic valve, annulus and LVOT from computed tomography were not available.

## Conclusion

VD-AR after THV implantation enables the operator to assess quantitatively regurgitation, to rationalise BPD and to assess its efficacy.
